# Analysis of neutrophil extracellular trap‐related genes in Crohn's disease based on bioinformatics

**DOI:** 10.1111/jcmm.70013

**Published:** 2024-08-28

**Authors:** Libin Chen, Feiyan Ai, Xing Wu, Wentao Yu, Xintong Jin, Jian Ma, Bo Xiang, Shourong Shen, Xiayu Li

**Affiliations:** ^1^ Department of Gastroenterology The Third Xiangya Hospital of Central South University Changsha China; ^2^ Hunan Key Laboratory of Nonresolving Inflammation and Cancer The Third Xiangya Hospital of Central South University Changsha China; ^3^ Department of Pathology, The Third Xiangya Hospital Central South University Changsha Hunan China; ^4^ The Key Laboratory of Carcinogenesis and Cancer Invasion of the Chinese Ministry of Education, Cancer Research Institute and School of Basic Medical Sciences Central South University Changsha China

**Keywords:** bioinformatics, biomarker, Crohn's disease, Gene Expression Omnibus, immune infiltration, neutrophil extracellular traps

## Abstract

Crohn's disease (CD) presents with diverse clinical phenotypes due to persistent inflammation of the gastrointestinal tract. Its global incidence is on the rise. Neutrophil extracellular traps (NETs) are networks released by neutrophils that capture microbicidal proteins and oxidases targeting pathogens. Research has shown that NETs are implicated in the pathogenesis of several immune‐mediated diseases such as rheumatoid arthritis, systemic lupus erythematosus and inflammatory bowel disease. The goal of this study was to identify a panel of NET‐related genes to construct a diagnostic and therapeutic model for CD. Through analysis of the GEO database, we identified 1950 differentially expressed genes (DEGs) associated with CD. Gene enrichment and immune cell infiltration analyses indicate that neutrophil infiltrates and chemokine‐related pathways are predominantly involved in CD, with other immune cells such as CD4 and M1 macrophages also playing a role in disease progression. Utilizing weighted gene co‐expression network analysis (WGCNA) and protein–protein interaction (PPI) networks, we identified six hub genes (SPP1, SOCS3, TIMP1, IRF1, CXCL2 and CD274). To validate the accuracy of our model, we performed external validation with statistical differences(*p* < 0.05). Additionally, immunohistochemical experiments demonstrated higher protein expression of the hub genes in colonic tissues from CD patients compared to healthy subjects (*p* < 0.05). In summary, we identified six effective hub genes associated with NETs as potential diagnostic markers for CD. These markers not only offer targets for future research but also hold promise for the development of novel therapeutic interventions for CD.

## 1 INTRODUCTION

Crohn's disease (CD) is a chronic, recurrent inflammatory disorder of the intestine that can affect any part of the digestive tract from the mouth to the anus, and it is also associated with extraintestinal complications. Symptoms include diarrhoea, abdominal pain, fever, weight loss and fatigue. In severe cases, CD may lead to complications such as intestinal obstruction, fistulas and abdominal abscesses, which often require surgical intervention and have a poor prognosis.^1^, ^2^ Although CD is typically diagnosed in patients under 30, its incidence is rising among elderly individuals. Certain populations, such as German Jews, those living in urban areas, and individuals residing in northern latitudes, have a higher incidence of CD.^3^ The disease is most common in people aged 20–40 years. In Asian populations, CD is more prevalent in men than in women.^4^, ^5^ The pathogenesis of CD is complex and not fully understood. Genetic susceptibility to the disease has been established, and specific environmental factors have been associated with its development.^2^, ^6^

Neutrophils are crucial components of the innate immune system, acting as the first line of defence against pathogens and tissue damage. Upon infection or injury, they are rapidly recruited to eliminate pathogens.^7^, ^8^ Neutrophil extracellular traps (NETs) are intricate structures released into the extracellular space upon neutrophil activation, playing a vital role in both innate and adaptive immunity. Initially discovered as a novel antimicrobial mechanism, recent studies suggest that NETs may also contribute to hyperinflammatory pathology in their own tissues.^9^, ^10^ Research on mouse models of colitis indicates that NETs can induce apoptosis in epithelial cells and disrupt tight and adherens junctions.^11^ Moreover, the density of NETs increases with the severity of histopathological changes in CD, with a significant positive correlation between the NET marker intensity and the severity of CD in samples.^12^

Previous research has yet to explore the gene mechanisms behind both CD and NETs using bioinformatics techniques. To address this gap, we conducted a study utilizing microarray datasets (GSE186582 and GSE179285) from the Gene Expression Omnibus (GEO) to identify differentially expressed genes (DEGs) between CD patients and healthy individuals. Our analysis included WGCNA to identify an upregulated module (cyan) related to CD inflammatory response, immune cell infiltration and protein–protein interaction (PPI) network analysis to identify hub genes, which were further validated using immunohistochemistry. The study aimed to uncover genes and pathways associated with CD and NETs, providing valuable insights for CD diagnosis and treatment.

## 2 MATERIALS AND METHODS

### 2.1 Data acquisition and ethical approval

The keyword ‘Crohn's disease’ was used to search for CD gene expression profiles in the GEO database (https://www.ncbi.nlm.nih.gov/geo/). To ensure accuracy and reliability, the following standard filters were applied: (1) CD sequencing results had to be obtained from the analysis of human colon samples; (2) The dataset must include both cases and controls, with each group containing more than six samples to ensure the robustness of the analysis; (3) The dataset must have a balanced distribution of data; (4) Patients must not have received immunosuppressive therapy 1 month prior to the sequencing analysis. Following these criteria, the GEO datasets numbered GSE165512, GSE186582 and GSE179285 were selected. The detailed description of the dataset can be found in Table 1. On the Microbiology platform (https://www.bioinformatics.com.cn/), we created heat maps of the datasets. We obtained a list of neutrophil extracellular trap genes (NRGs) from a previous study.^13^ As this study utilized previously collected clinical samples, did not include any identifying information, and did not pose any risks to the participants, obtaining informed consent was not deemed necessary. This study was approved by the Ethics Committee of the Third Xiangya Hospital, Central South University (Reference Number: 2023220), ensuring that it adhered to ethical standards.

**TABLE 1:** Information from three Crohn's disease‐related datasets from the GEO database.

GEO number [year]	Crohn's disease	Healthy control	Sample type	Platforms	Description
GSE186582 [2021]	196	25	Colon biopsies	GPL570	Affymetrix Human Genome U133 Plus 2.0 Array
GSE179285 [2021]	47	31	Colon biopsies	GPL6480	Agilent‐014850 Whole Human Genome Microarray 4 × 44K G4112F
GSE165512 [2021]	84	46	Colon biopsies	GPL16791	Illumina HiSeq 2500 (Homo sapiens)

### 2.2 Defining DEGs and functional enrichment analysis

We used the GEO2R online tool (https://www.ncbi.nlm.nih.gov/geo/geo2r/) to identify DEGs with a cut‐off criterion of adjusted *p* < 0.05 and logFC > 0. We visualized the overlap of DEGs using an online Venn diagram generator (http://bioinformatics.psb.ugent.be/webtools/Venn/). To investigate the functions of these DEGs, we performed gene ontology (GO) analyses using the ‘clusterprofiler’ R package, which included biological processes (BP), cellular components (CC) and molecular functions (MF), and mapped the results using the ‘ggplot2’ R package. Additionally, we conducted Kyoto Encyclopedia of Genes and Genomes (KEGG) pathway enrichment analysis and performed further analysis on the DAVID website (https://david.ncifcrf.gov/), followed by visualization on the Microbiology platform (https://www.bioinformatics.com.cn/) using bubble maps. The significance cut‐off was set to *p* < 0.05.

### 2.3 Immune cell infiltration analysis

CIBERSORT is a method for calculating cell composition based on expression profiles.^14^ The deconvolution algorithm is used to calculate the abundance of 22 immune cells (LM 22) in CD patients and healthy individuals in the dataset GSE179285. The sum of the 22 immune cell type scores in each sample was 1. Heat maps of immune cell abundance were performed by ‘ggplot2’ R, and box plots were drawn by the ‘ggpubr’ R package. Furthermore, we evaluated the degree of infiltration of 28 immune cell types using gene expression levels within 28 published immune cell gene sets by applying the single sample gene set enrichment analysis (ssGSEA) method in the R package GSVA.^15^, ^16^

### 2.4 Building weighted correlation networks

We performed a weighted gene co‐expression network analysis (WGCNA) on the gene expression data from GSE179285 using the ‘WGCNA’ package in R.^17^ The analysis involved several steps, including sample clustering to identify and exclude outlier samples, construction of co‐expression networks and module identification and identification of inflammation‐associated modules. We then used Venn diagrams to identify the intersection of genes from the inflammatory modules with DEGs and NRGs.

### 2.5 Generating protein–protein interaction networks and identifying hub genes

We utilized the STRING database (https://string‐db.org/) to establish PPI networks of the overlapping genes. Subsequently, we imported these overlapping genes into Cytoscape v3.9.0 and used the DMNC algorithm in Cytohubba for the identification of hub genes.

### 2.6 External validation

To verify the findings of our study, we downloaded the GSE165512 dataset from the GEO database, which comprises biopsies from 84 patients with CD and samples from 46 healthy individuals. In order to determine threshold values, we assessed the expression differences between the two groups by plotting receiver operating characteristic (ROC) curves of the hub genes using R, and AUC was calculated to evaluate the clinical diagnostic significance of the key genes.

### 2.7 Analysis of immunohistochemical results

To verify the differential expression of central genes in colonic tissue from individuals with CD and healthy subjects, immunohistochemical staining was conducted. Archived colon tissue samples from 10 CD patients who had undergone colonoscopy as well as four healthy subjects at the Third Xiangya Hospital of Central South University were used for clinical testing. The paraffin sections were prepared using 10V4 and dried. Xylene and ethanol solutions were used to deparaffinize them. Heat‐induced antigen repair and blocking were carried out using citrate buffer, followed by two rinses with PBS buffer. Primary antibodies, including SPP1 (340690, ZenBio), IRF1 (R24756, ZenBio), SOCS3 (bs‐0580R, Bioss), CXCL2 (bs‐1162R, Bioss), TIMP1 and CD274, were incubated overnight at 4°C. Three times with PBS buffer, wash the sections, secondary antibodies were applied for 20 min at room temperature, and the sections were stained with 3,3′‐diaminobenzidine and haematoxylin. For each index, 40× high magnification fields were randomly observed, and ImageJ software was used to calculate the mean optical density (MOD) values.

### 2.8 Analyses of statistics

The statistical software GraphPad Prism 8.0.2 and R 4.1.2 were used to analyse and visualize the data. *t*‐tests were used to calculate differences between CD samples and healthy samples in central gene expression. *p* < 0.05 was considered statistically significant (*p < 0.05, **p < 0.01, ***p < 0.001).

## 3 RESULTS

### 3.1 Identification and functional enrichment analysis of DEGs

**FIGURE 1:**
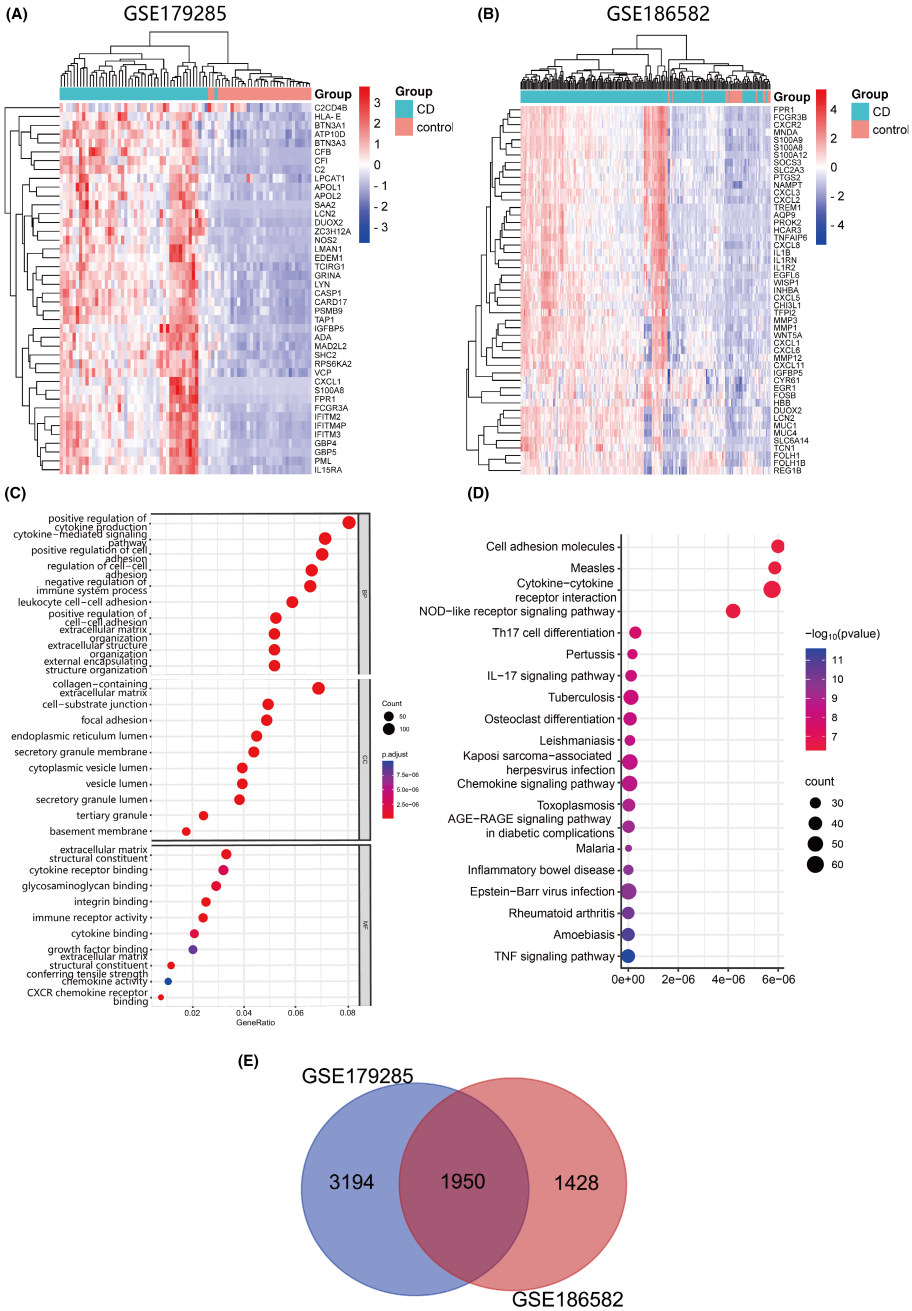
Functional enrichment analysis and Venn diagram of DEGs: (A, B) Heatmap of the expression of the top 50 DEGs in control and CD samples in GSE186582 and GSE179285. (C) GO analysis for DEGs. (D) KEGG analysis for DEGs. (E) Venn diagram demonstrating DEGs in the two datasets (GSE186582 and GSE179285).

We used the GEO2R online tool to analyse two CD‐related datasets (GSE186582 and GSE179285). We filtered genes with an adjusted *p* < 0.05 and logFC > 0, and the top 50 DEGs for both datasets were presented in a heat map (Figure 1A,B). To identify the most significant DEGs, we generated Venn diagrams, identifying 1950 intersecting DEGs (Figure 1E). Following this, we conducted GO and KEGG enrichment analyses on these DEGs. The results, depicted in Figure 1C, highlight the top 10 significantly enriched GO terms, categorizing them into MF, CC and BP. Concurrently, the KEGG analysis, shown in Figure 1D, identifies the top 20 most significantly enriched pathways. Our findings suggest that the BP of these DEGs are predominantly linked to the promotion of cytokine production. Moreover, the KEGG enrichment analysis underscores the significant involvement of immune‐related pathways, including the TNF signalling pathway, chemokine signalling pathway, Th17 cell differentiation and cytokine and cytokine receptor interactions.

### 3.2 WGCNA and modular analysis

**FIGURE 2:**
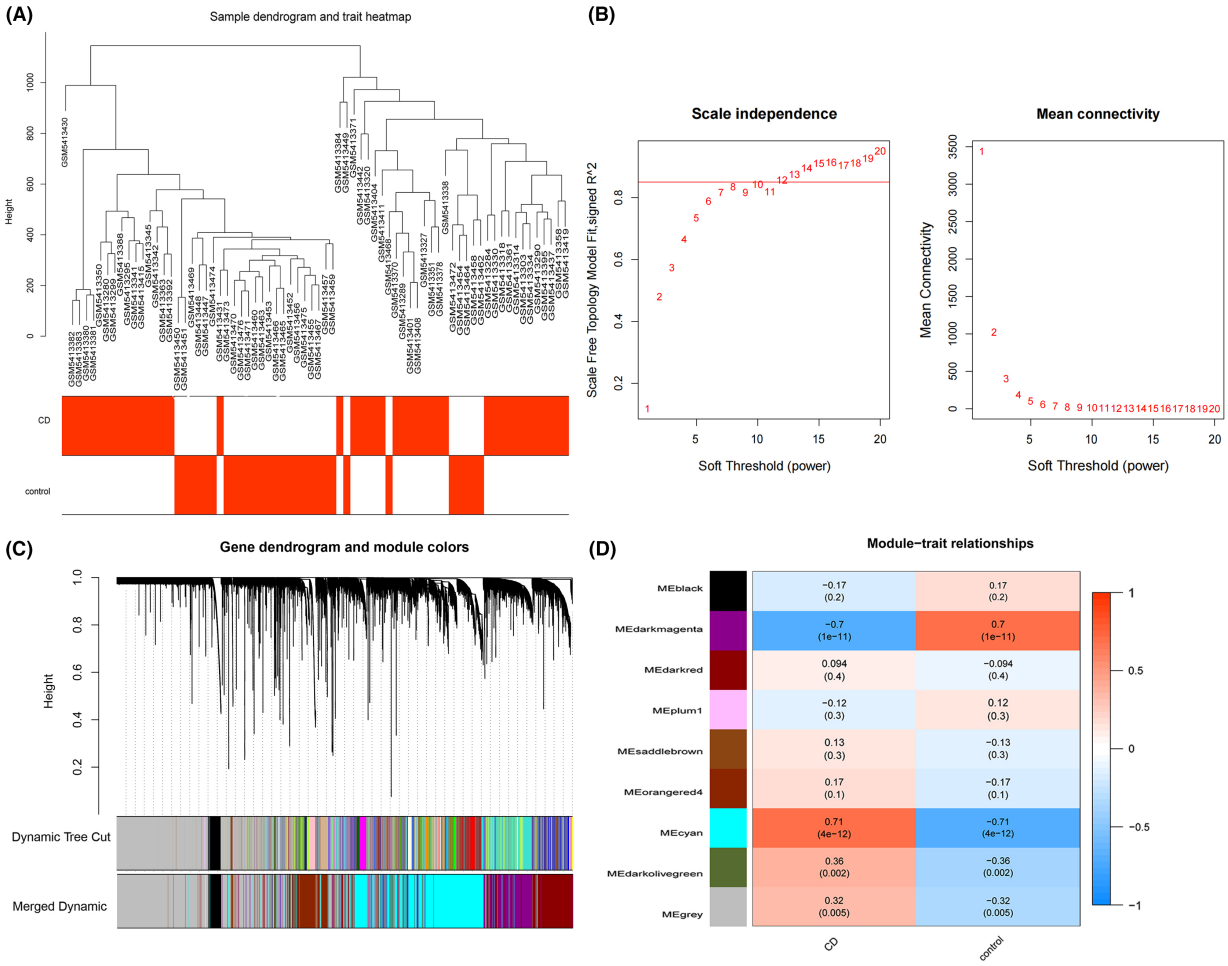
WGCNA analysis of dataset GSE179285: (A) Cluster analysis of samples. (B) Soft threshold non‐scale fitting index analysis and soft threshold average connectivity analysis. (C) Gene dendrogram and module colours based on gene expression patterns. (D) Module correlation analysis.

Utilizing the ‘WGCNA’ R package, we constructed a weighted gene co‐expression network based on the expression profiles of the GSE179285 dataset. Prior to constructing the network, we conducted clustering analysis on the samples and found no significant heterogeneity between them. The samples were subsequently labelled with two clinical parameters, inflammation and healthy individuals, to extract inflammation‐related phenotypic features (Figure 2A). To establish the scale‐free network, we set the soft threshold to *β* = 12, the scale independence value to 0.85 (Figure 2B), and ensured that the correlation coefficient greater than 0.8 was achieved between log(k) and log(p(k)). We identified a total of eight independent modules, with grey modules indicating genes that could not be clustered into any module (Figure 2C). We then visualized the correlation between modules and clinical features (Figure 2D). Based on the *p*‐value ranking, we selected the cyan module, which was upregulated and most relevant to inflammation. This module contained 5507 genes identified as inflammation‐related.

### 3.3 Comparison of immune infiltration

**FIGURE 3:**
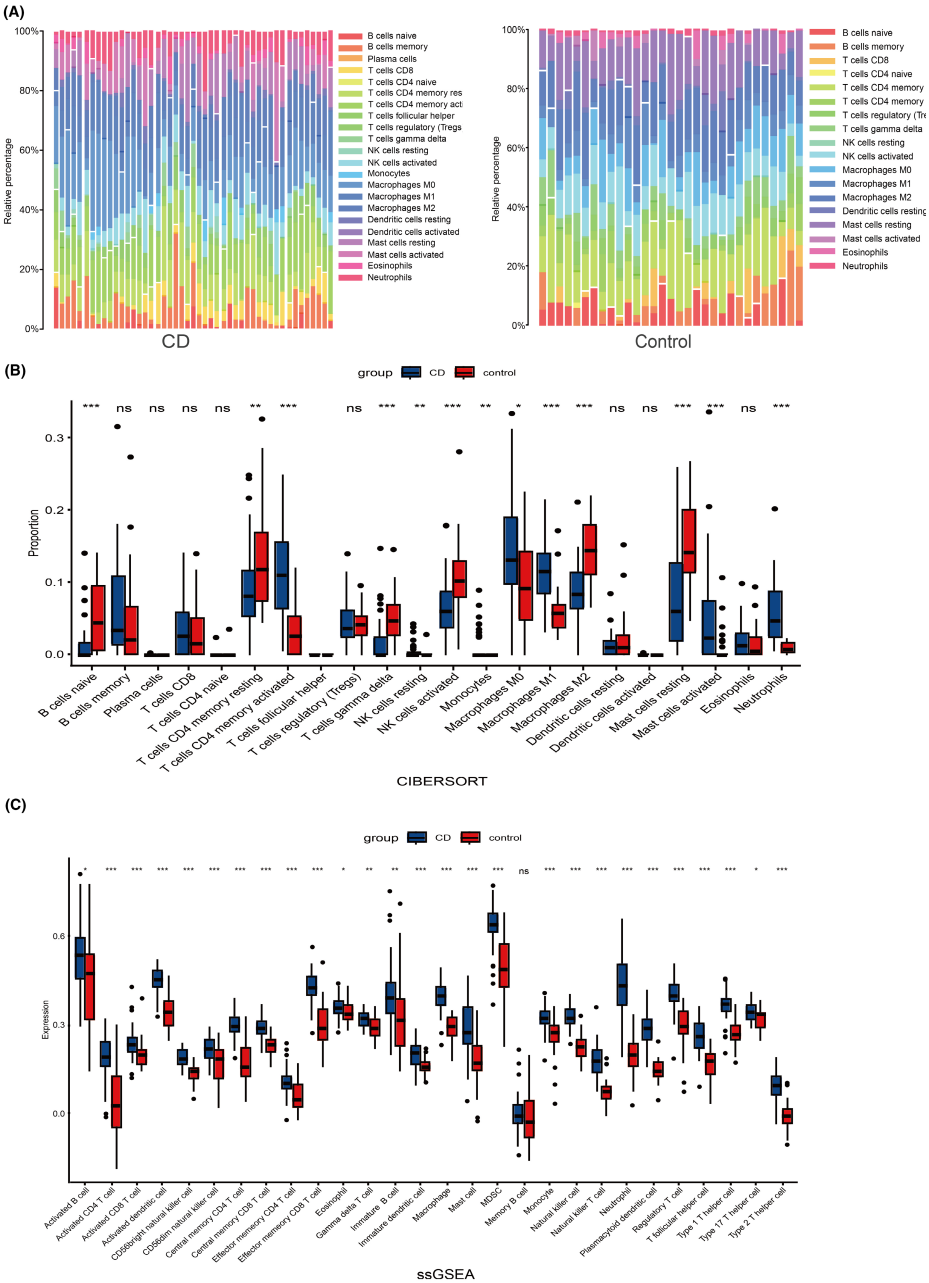
Comparison of immune characteristics between CD and control. (A) Heatmap for immune cells of the CD and control. (B) cibersort (proportion of immune cells). (C) ssGSEA (expression of immune cells). The *p* values are labelled using asterisks (ns, no significance, *p < 0.05, **p < 0.01, ***p < 0.001).

We performed CIBERSORT and ssGSEA analyses on the GSE179285 dataset to deepen our understanding of immune cell infiltration in CD. The CIBERSORT analysis provided insights into the composition of immune cells, indicating a marked increase in neutrophils, activated CD4 memory T cells, M1 macrophages and activated mast cells in CD patients compared to healthy controls (Figure 3A,B). This suggests a heightened inflammatory response characteristic of CD. Furthermore, the ssGSEA analysis identified elevated expression levels of 27 immune cell subtypes in CD patients. Notably, these included neutrophils, activated CD8 T cells, myeloid‐derived suppressor cells, natural killer cells, natural killer T cells and activated CD4 T cells (Figure 3C). These findings provide a comprehensive profile of the immune landscape in CD, highlighting the diverse array of immune cells involved in the disease's pathogenesis.

### 3.4 Hub gene identification

**FIGURE 4:**
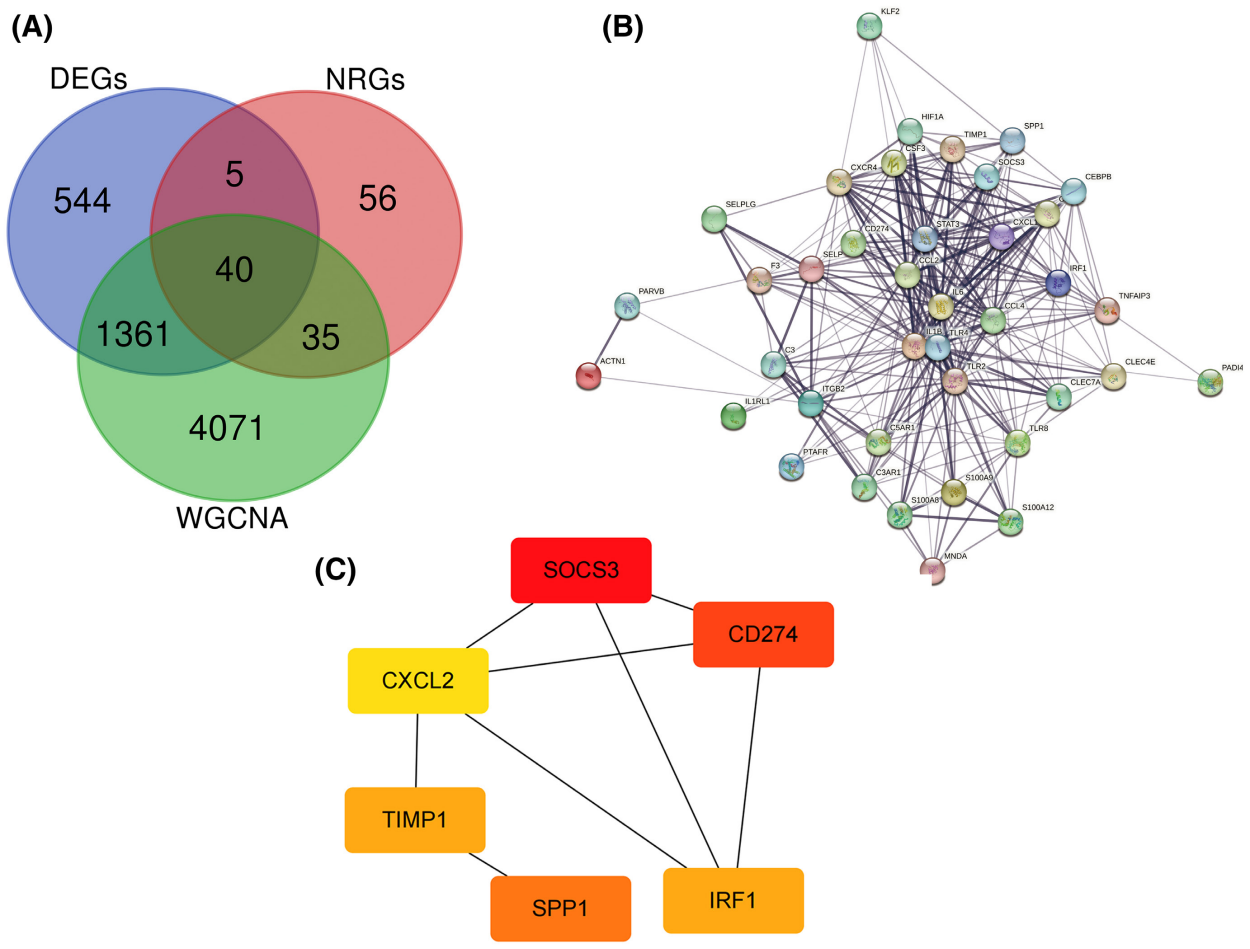
Hub gene identification: (A) Venn diagram of DEGs, NRGs and cyan module genes. (B) PPI network constructed with the 40 CD‐related genes. (C) Results of DMNC algorithm from Cytohubba (top six genes).

Following KEGG and GO enrichment analyses and immune cell infiltration studies, it was evident that CD is predominantly driven by the activation of inflammatory cytokine signalling and neutrophil activation. These analyses highlighted that the inflammatory pathways are significantly upregulated in CD, leading to the recruitment and activation of immune cells, particularly neutrophils, which play a critical role in the disease's pathology. Recent studies have further implicated NETs in the pathogenesis of CD, suggesting that NET formation may exacerbate inflammation and tissue damage in the gut. Consequently, we focused on identifying genes associated with CD inflammation. By overlapping the DEGs from our previous analyses with NET‐related genes (NRGs) and 5507 inflammation‐related genes identified through WGCNA of the GSE179285 dataset, we narrowed down a list of 40 genes relevant to CD inflammation (Figure 4A). To gain deeper insights into the interactions among these genes, we conducted a PPI network analysis using the STRING database. The PPI data were then imported into Cytoscape software for visualization and further analysis. Utilizing the cytoHubba plug‐in with the DMNC algorithm, we identified the top six hub genes: SPP1, SOCS3, TIMP1, IRF1, CXCL2 and CD274 (Figure 4B,C). These hub genes are likely to play pivotal roles in the inflammatory processes underlying CD.

### 3.5 External validation of hub genes

**FIGURE 5:**
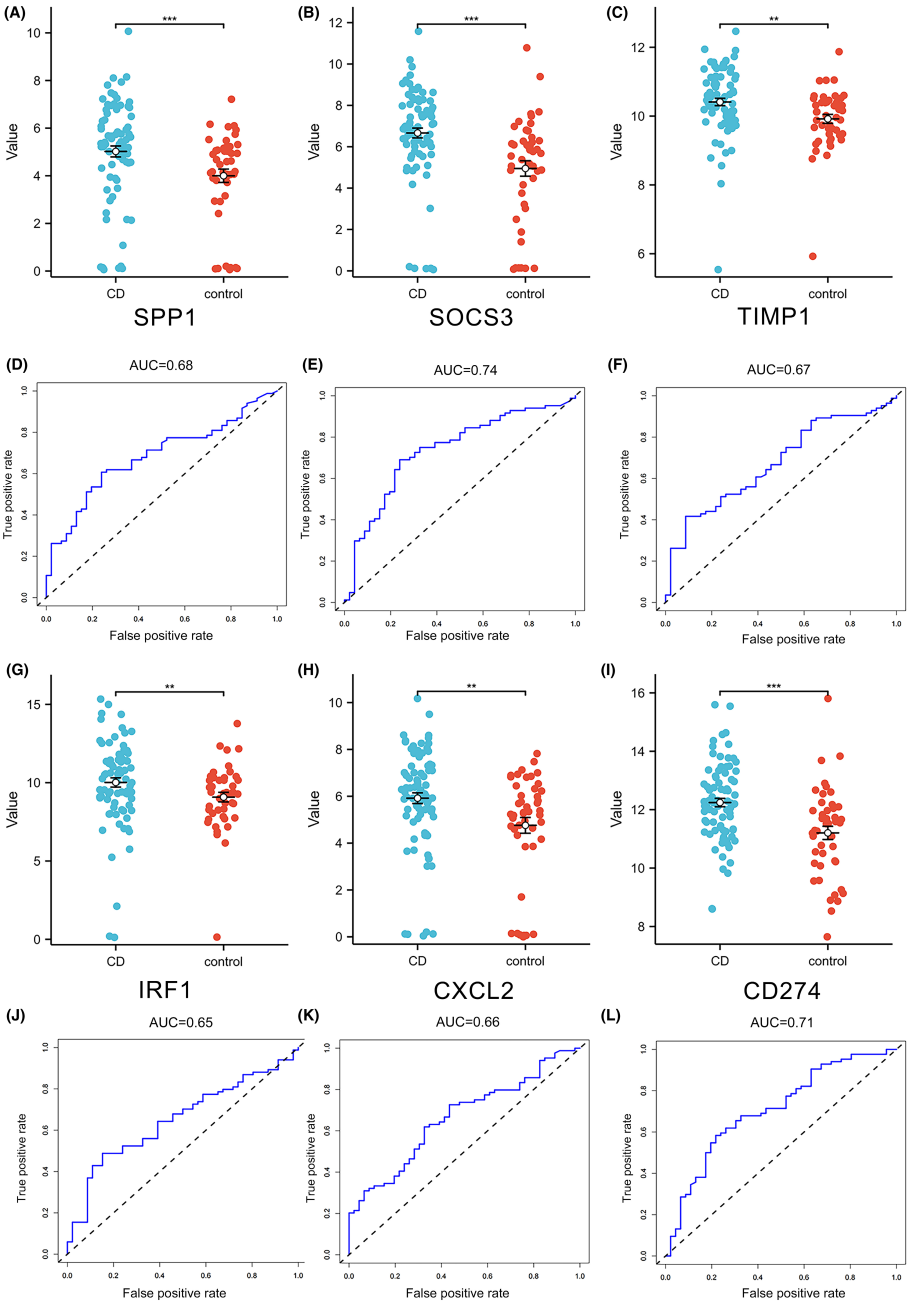
External validation of hub genes. (A–F) Hub gene expression between Crohn's disease group and control group. (G–L) the ROC curve of the hub gene. The *p* values are labelled using asterisks (ns, no significance, **p < 0.01, ***p < 0.001).

To validate our findings, we cross‐referenced our results with another dataset (GSE165512) from the GEO database. This comparison revealed that patients with CD expressed significantly higher levels of six specific genes (SPP1, SOCS3, TIMP1, IRF1, CXCL2 and CD274) in their colonic tissues compared to healthy subjects, reinforcing our initial results (Figure 5A–F). We also evaluated the potential of these genes as biomarkers for CD by performing ROC curve analysis on colonic tissue samples from both CD patients and healthy individuals. The ROC curve analysis indicated that the AUC values for SPP1, SOCS3, TIMP1, IRF1, CXCL2 and CD274 were all above 0.65 (Figure 5G–L). Additionally, the AUC values for SPP1, SOCS3 and TIMP1 in another dataset (GSE95095) were 0.847, 0.934 and 0.958, respectively (Figure S1A). Overall, our results suggest that these six genes are significantly associated with CD and hold potential as diagnostic biomarkers.

### 3.6 Immunohistochemistry validation of potential biomarkers

**FIGURE 6:**
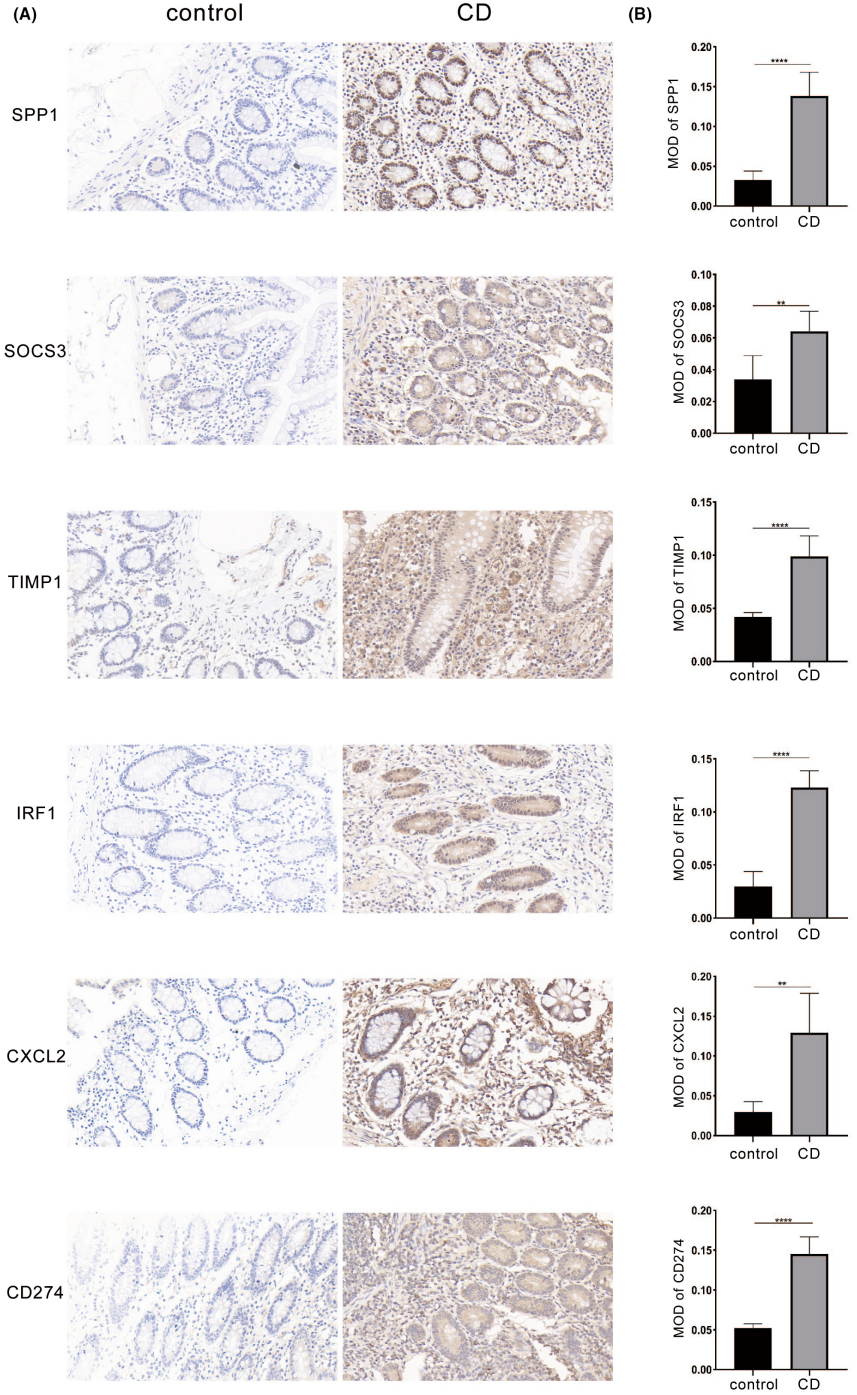
The expression levels of the identified hub genes were evaluated in both Crohn's disease patients and control group. Immunohistochemical analysis revealed that the expression of the hub genes was significantly higher in the disease group compared to the control group, with all *p*‐values <0.05, indicating statistical significance (A, B). The *p* values are labelled using asterisks (ns, no significance, **p < 0.01, ****p < 0.001).

Overall, our results suggest that these six genes are significantly associated with CD and hold potential as diagnostic biomarkers. Among the six hub genes identified, the subcellular localization varies: SPP1 primarily resides in the nucleus, SOCS3 and TIMP1 are mainly present in the cytoplasm, IRF1 is observed in both the nucleus and cytoplasm, CXCL2 is distributed in the cytoplasm and extracellular matrix and CD274 is found in the cell membrane and cytoplasm. The positive expression of these genes was indicated by brown or gold colouration in immunohistochemical analysis. To validate the expression of these proteins, we performed immunohistochemical analysis on colon tissues from CD patients and healthy individuals. Our findings revealed significantly higher MOD values for SPP1, SOCS3, TIMP1, IRF1, CXCL2 and CD274 proteins in colon tissues from CD patients compared to healthy individuals (all *p* < 0.05), confirming the upregulation of these proteins in CD patients and validating our bioinformatics analysis (Figure 6A,B).

## 4 DISCUSSION

With the advancements of biologic and immunosuppressive agents, patients with CD are now experiencing significantly improved survival rates.^18^ Despite these advancements, diagnosing CD remains challenging due to the rate of delayed diagnoses. CD diagnosis involves a comprehensive evaluation of symptoms, clinical signs, endoscopy and imaging, making accurate differential diagnosis essential. The development of new biomarkers may facilitate early diagnosis and targeted treatment of CD. This research utilized GO and KEGG enrichment and immune cell infiltration analyses on two CD‐associated GEO datasets from colonic biopsy samples to explore the molecular signatures involved in CD. Our findings identified 5507 genes associated with CD inflammation through WGCNA analysis. By cross‐referencing with NET‐associated genes and constructing PPI networks, we identified SPP1, SOCS3, TIMP1, IRF1, CXCL2 and CD274 as potential diagnostic markers. These markers were validated through immunohistochemical analysis. In this discussion, we aim to explore the significance of their expression patterns and their potential as diagnostic markers for CD.

CIBERSORT immune infiltration analysis revealed significant upregulation of neutrophils, memory‐activated CD4^+^ T cells, M1‐type macrophages and activated mast cells in CD patients. Research indicates that one pathogenic mechanism of CD involves the compromised intestinal mucosal barrier and substantial microbial invasion.^19^ In cases where adaptive immunity is insufficiently activated, the innate immune system is rapidly activated by the intestinal contents, and the lamina propria macrophages,^20^ mast cells^21^ and lymphocytes^22^ rapidly release large amounts of pro‐inflammatory cytokines to combat microbial infections. The pro‐inflammatory cytokines also induce microvascular changes leading to a massive recruitment of neutrophils to eliminate the invading bacteria.^23^, ^24^ These results align with our immune infiltration analysis.

Neutrophils play a critical role in intestinal injury in IBD,^25^ the decrease in neutrophil recruitment and delay in bacterial clearing were found at sites of inflammation in the intestine and at sites of systemic injury in CD,^26^ suggesting that the impaired innate immune responses in CD may result in chronic inflammation and granuloma formation.^27^ Neutrophils contribute to inflammation by forming NETs, which consist of DNA scaffolds with histones and proteins from cytotoxic neutrophils, released to contain microbes during infection and inflammation.^28^, ^29^ Based on GO, KEGG and immune infiltration analyses, we selected hub genes by cross‐referencing with previously reported NET‐associated genes.

Studies show that NETs are more prevalent in the inflamed mucosa, faeces or blood of IBD patients.^30^ The abundance of NETs correlates with active disease^12^ and key NET proteins, including neutrophil elastase (NE) and myeloperoxidase (MPO),^31^, ^32^ which are highly specific for neutrophils and are involved in the depolymerisation of chromatin during the formation of NETs, have been found to be increased in IBD through liquid chromatography–mass spectrometry‐based proteomics studies. Immunohistochemical analysis of CD patient samples also revealed a significant increase in the presence of NET markers NE, MPO and citrullinated histone (CitH3) with increasing histopathology score.^12^ Therefore, identifying key biomarkers in NET‐associated genes through bioinformatic approaches may provide valuable insights for developing new immunotherapies for the inflammatory response in CD.

We utilized the STRING platform to establish a PPI network and identified the top six central genes associated with NETs among the differentially expressed inflammation genes in CD. Using Cytoscape for network analysis, we determined that these central genes were SPP1, SOCS3, TIMP1, IRF1, CXCL2 and CD274. The differential expression of these genes in CD tissues, compared to healthy controls, strongly suggests their involvement in the pathogenesis of CD. SPP1, also known as osteopontin (OPN), was found to be expressed in the terminal ileum of CD patients.^33^, ^34^, ^35^, ^36^ Notably, an eight‐single‐nucleotide polymorphism haplotype in the OPN gene is significantly associated with CD susceptibility.^37^ Additionally, OPN was shown to support Th17 differentiation in a mouse colitis model,^38^ which aligns with the results of our KEGG enrichment analysis. OPN plays a pivotal role in the formation and regulation of NETs, potentially binding to histones, thereby impeding TLR receptor activation and inhibiting NET formation.^39^ During episodes of inflammation, OPN levels surge, countering the cytotoxic effects of extracellular histones. Cumulatively, the suppressive action of OPN on NET formation emerges as crucial in mitigating sustained tissue damage and remodelling throughout prolonged inflammation. This underscores the potential significance of SPP1 in the aetiology and progression of CD, particularly through their influence on the formation and functionality of NETs. Furthermore, induction of the transcription factor IRF1 by TNFα in IEC in vitro has been demonstrated to inhibit OPN expression.^40^ IRF1 serves as an interferon regulator that acts as a transcription factor in numerous BP.^41^, ^42^ A study observed increased IRF1 expression in 72% of CD patients in the study cohort, suggesting its potential contribution to inflammation in CD.^43^ IRF‐1 has been identified as a regulator of the classical ROS‐dependent NETosis mechanism, promoting NET production.^44^ This implies that IRF1 might be implicated in the development and progression of CD by modulating neutrophil activity and function, thereby influencing the regulation of NET formation and release. The expression of suppressor of cytokine signalling 3 (SOCS3), an inhibitor of cytokine signalling, has been consistently found to have increased expression in the inflamed colonic regions of patients with UC and CD. Several studies suggest that elevated SOCS3 expression in intestinal epithelial cells (IEC) correlates with a shorter time before relapse, implying a pathogenic role in UC relapse.^45^ Additionally, high SOCS3 expression may impede mucosal healing following mild inflammatory injury. Overexpression of SOCS3 inhibits c‐Myc induction, which can impair the mitogenic effects of IL‐22, thus hampering IL‐22‐mediated IEC proliferation and mucosal healing.^46^ Therefore, reducing high SOCS3 expression in CD patients may represent a viable strategy for clinical management.

CD274, also known as PDL1, serves as a ligand for programmed cell death protein 1 (PD‐1). The interaction between CD274 and its receptor inhibits T‐cell activation and cytokine production.^47^ Research has elucidated that the upregulation of PD‐L1 in neutrophils plays a significant role in regulating autophagy and facilitating the release of NETs through the PI3K/Akt/mTOR pathway.^48^ Furthermore, histopathological analysis of tumour patients with colitis during anti‐PDL1 monotherapy has revealed colonic tissue displaying neutrophil crypt microabscesses, significant crypt epithelial cell apoptosis and crypt atrophy/shedding, resembling inflammatory bowel disease.^49^ However, the exact mechanism remains unclear. These findings suggest the promising potential of targeting PDL1/PD‐1 for the treatment of IBD, an area that requires further exploration. TIMP1, a metalloproteinase inhibitor, binds to matrix metalloproteinases and hinders the synthesis and secretion of proteases. This action reduces collagen destruction and plays a critical role in the maintenance of the integrity of the intestinal barrier.^50^, ^51^ Elevated levels of TIMP1 protein have been observed in inflammatory and fibrotic lesions of CD.^52^ TIMP1 deficiency results in differential expression of immune‐related genes and attenuation of fibrosis development.^53^ These reports indicate that TIMP1 contribute to tissue damage and remodelling in CD. Understanding the role of TIMP1 in intestinal remodelling is crucial for developing more effective and targeted therapeutic strategies to combat the fibrosis of the bowel in CD. C‐X‐C motif chemokine ligand 2 (CXCL2) is expressed at inflammatory sites and plays a role in promoting neutrophil degranulation and chemotaxis through its receptor CXCR2 activation. In a mouse model of TNBS colitis, intervention with the CXCR2 inhibitor SB225002 significantly reduced tissue damage, inflammation and levels of IL‐1β, MIP‐2, iNOS and KC, thereby improving survival.^54^ Consistently, CXCR2 knockout mice exhibited suppressed colonic inflammation during DSS‐induced colitis, with reduced neutrophil infiltration and downregulation of the NET marker MPO.^55^ These findings demonstrate that targeting CXCL2/CXCR2 may serve as an effective approach for treating CD.

This study has a few limitations. First, there were objective reasons, such as experimental conditions, for the small clinical sample size. Additionally, the validation study was limited to a small‐scale immunohistochemistry experiment conducted at a single centre. Lastly, the analysis of relevant signalling pathways and central genes was only confined to bioinformatic analysis, and further experimental validation was necessary to corroborate the findings.

## 5 CONCLUSION

In summary, our study employed a bioinformatics approach to identify specific genes associated with NETs in the context of CD. We further elucidated the pathways and mechanisms through which these genes operate, shedding light on the potential involvement of NETs in CD. The findings from our investigation unveil novel targets that hold promise for the diagnosis and treatment of CD, offering potential clinical value in managing this condition.

## AUTHOR CONTRIBUTIONS

**Libin Chen:** Conceptualization (equal); data curation (equal); investigation (equal); visualization (lead); writing – original draft (equal). **Feiyan Ai:** Data curation (equal); methodology (equal); writing – review and editing (equal). **Xing Wu:** Data curation (equal). **Wentao Yu:** Data curation (equal); methodology (equal). **Xintong Jin:** Investigation (equal). **Jian Ma:** Writing – review and editing (equal). **Bo Xiang:** Writing – review and editing (equal). **Shourong Shen:** Supervision (equal). **Xiayu Li:** Conceptualization (equal); supervision (equal); writing – original draft (equal); writing – review and editing (equal).

## FUNDING INFORMATION

This work was supported in part by grants from The National Natural Science Foundation of China (82172766), the Natural Science Foundation of Hunan Province, China (2020JJ4838), The Scientific Research Project of Hunan Provincial Health Commission (20201040).

## CONFLICT OF INTEREST STATEMENT

The authors declare that they have no conflicts of interest.

## Supporting information


Figure S1.

## Data Availability

The data that support the findings of this study are available in NCBI at https://www.ncbi.nlm.nih.gov/geo/. These data were derived from the following resources available in the public domain: GSE165512, GSE186582, and GSE179285.
