# Supplementary figures and images for: Analysis of neutrophil extracellular trap‐related genes in Crohn's disease based on bioinformatics
Source: J Cell Mol Med. 2024 Aug 28;28(16):e70013. doi: 10.1111/jcmm.70013 (PMC11358036; doi:10.1111/jcmm.70013)

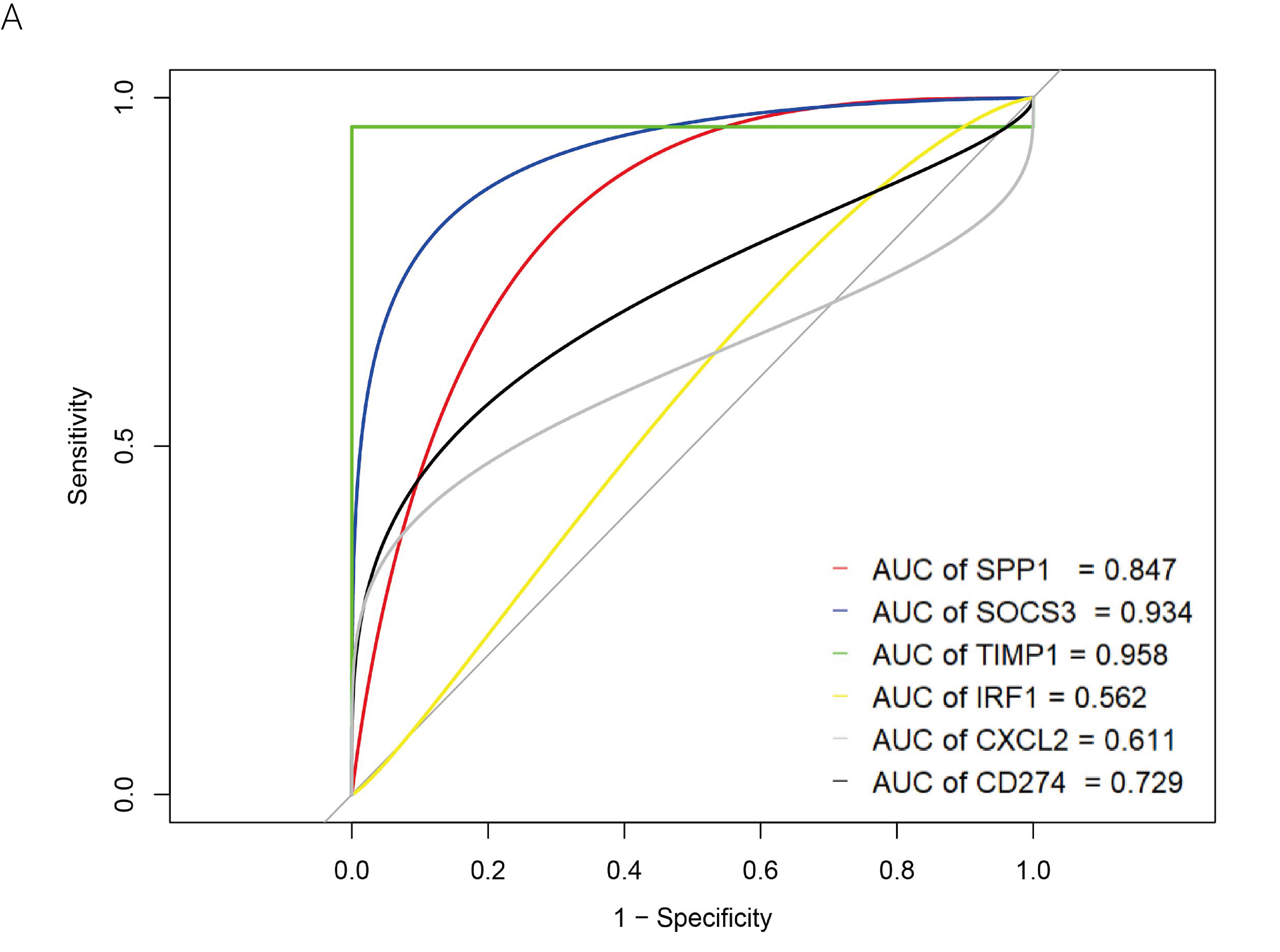

Supplement: Supplementary file 1 — Figure S1. [file JCMM-28-e70013-s001.jpg]
